# Supplementary material for: Early warning signals for predicting cryptomarket vendor success using dark net forum networks
Source: Sci Rep. 2024 Jul 16;14:16336. doi: 10.1038/s41598-024-67115-5 (PMC11251263; doi:10.1038/s41598-024-67115-5)
Supplement: Supplementary file 1 — Supplementary Information. [file 41598_2024_67115_MOESM1_ESM.pdf]

Supplementary Material
Early warning signals for predicting cryptomarket vendor success using dark net forum networks

Hanjo D. Boekhout[1,*], Arjan A.J. Blokland[2,3], and Frank W. Takes[1]

[1]Leiden University, Institute of Advanced Computer Science, Niels Bohrweg 1, 2333 CA Leiden, Netherlands
[2]Leiden University, Institute of Criminal Law and Criminology, Steenschuur 25, 2311 ES Leiden, Netherlands
[3]Netherlands Institute for the Study of Crime and Law Enforcement (NCSR), De Boelelaan 1077, 1081 HV Amsterdam, Netherlands
[*]h.d.boekhout@liacs.leidenuniv.nl

S1 Robustness of results

The results presented in the main paper rely on the network extracted based on a single set of parameter values. Here, we explore the robustness of those results for different parameter values (see Methods for a description of these parameters). We do so by changing the value of a single parameter at a time while maintaining the same values for all other parameters. Figures S1 and S2 shows vendor recall trends (similar to main paper Figure 2a) for each set of parameter values. Each row of plots in these figures shows the results for changing one specific parameter, with the middle column corresponding to the 'default' values. Note that since we are only changing the network formation process, the vendor recall results for the forum activity indicators do not change. As such, they provide a visual aid in analysing the changes in network measure performance.

Figure S1 shows vendor recall trends for varying values for the parameters influencing the formation of edges. First, δ_o determines how many posts two posts may be apart at most, to still form an edge connecting the users who placed them. Figures S1a–e show that the in-degree performs better at lower values of δ_o. Similarly, bidirectional harmonic closeness centrality performs slightly better at lower values. However, even then neither comes close to outperforming betweenness centrality. Some variation in performance can be observed for betweenness centrality as well. Slightly better performance, than default, are observed for $\delta_o = 5$ and 20. As such, there is no indication that specifically using a smaller or larger value of δ_o would improve performance. Second, δ_t determines how much time between two posts may have elapsed at most, to still form an edge connecting the users who placed them. Figures S1f–j show that changing δ_t between 7 days and 3 months hardly affects the vendor recall at all. Thus, our findings in the main paper can be considered robust for both δ_o and δ_t.

Figure S2 shows vendor recall trends for varying values for the parameters influencing the weight of edges. First, ω_{lower} and t_{lim} determine the scope and rate of decay of the exponential weighting function applied to "regular" edges, i.e., the implied social ties. ω_{lower} sets a minimum weight, and t_{lim} determines after how much time (between the placement of posts) this minimum weight is reached. For both of these parameters we can see no meaningful change in the vendor recall performance for either lower or higher values (see Figures S2a–j). As such, our findings in the main paper can be considered robust for both ω_{lower} and t_{lim}. Second, ω_{first} sets the standard weight for all edges formed by linking to the initial post of topics. Figures S2k–o show slight improvement of vendor recall for weighted directed PageRank as this parameter gets closer to one, the maximum weight. However, it remains the case that at no point does it outperform betweenness centrality. We also observe a slight variation in the performance of betweenness centrality. Specifically, we see movement in when it performs well. It appears that betweenness centrality performs slightly better in the early months for lower values of ω_{first}, whereas it performs slightly better in the later months for higher values. As such, it may be advantageous to choose the ω_{first} value based on the age of the cryptomarket one is dealing with. Regardless, the change in performance is small and we can conclude that our findings in the main paper can also be considered robust for ω_{first}.

Having established the robustness of changing a single parameter at a time, we finally consider changing several parameters simultaneously. For this we first combined parameter values which showed slight improvements compared to default parameter values in Figures S1 and S2 and plot the vendor recall trends for various combinations of these values in Figure S3a–j. Although some combinations show relatively improved performance for the centrality measures, none show sufficient improvement to become inconsistent with our findings.

Next, we investigate how potential loss of information caused by parameters δ_o and δ_t, as described in the "Network extraction" section of the main paper, impacts vendor recall performance if chosen differently in Figures S3k–n (i.e., the bottom row of Figure S3). Specifically, we consider each variation of including fewer or more posts and allowing for less or more

maximum time between posts. The figures show that the performance changes are largely dominated by the δ_o parameter, showing the same performance changes as those we observed for the default δ_t. Specifically, we see a slight improvement in the performance of betweenness centrality at both a lower and higher value of δ_o than default. Thus, there does not seem to be a clear connection between the performance and the information loss caused by smaller δ_o values. On the contrary, Figures S3k,m show that when we decrease δ_t to 14 days, the performance drops slightly for all tested δ_o parameter values. However, Figures S3l,n show that when we extend δ_t to 3 months, we observe the same performance as at 1 month for the same δ_o parameter value. As this was also the case for the default value of δ_o, we may conclude that although performance can be compromised by the information loss caused by δ_t, there is a point beyond which extending δ_t no longer improves performance. In other words, there is a value of δ_t at which the performance plateaus. It appears that for the Evolution cryptomarket this aligns with our chosen default value of 1 month. However, note that even at a δ_t as low as 7 days (Figure S1f), the performance drop observed is very small. Thus, it appears that the vast majority of the added-value to performance lies in the responses that are written within days of a post.

In short, we observe that in general the vendor recall performance varies little for changes in parameter values determining the formation of the network. Thus, we can consider our findings in the main paper to be robust for these parameters.

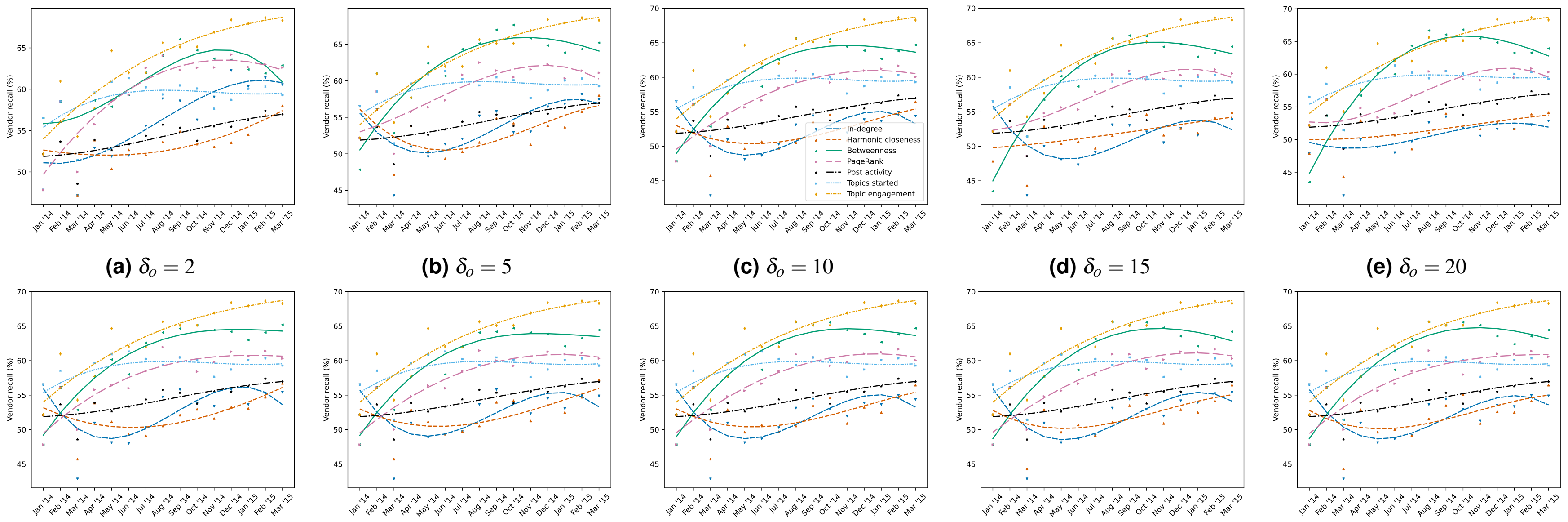

(a) $\delta_o = 2$ **(b)** $\delta_o = 5$ **(c)** $\delta_o = 10$ **(d)** $\delta_o = 15$ **(e)** $\delta_o = 20$

(f) $\delta_t = 7$ days **(g)** $\delta_t = 14$ days **(h)** $\delta_t = 1$ month **(i)** $\delta_t = 2$ months **(j)** $\delta_t = 3$ months

Figure S1. Results for varying edge formation parameters. Monthly vendor recall of top vendor percentile (top 0-20% vendors in terms of sales) among the top 20% of all users based on the network measures and activity indicators for current success. Each plot displays monthly vendor recall for a different set of parameter values used for generating the network. Each row of plots varies a single parameter influencing edge formation, with the remaining parameters at their default values. The center column always corresponds exactly with the default parameters.

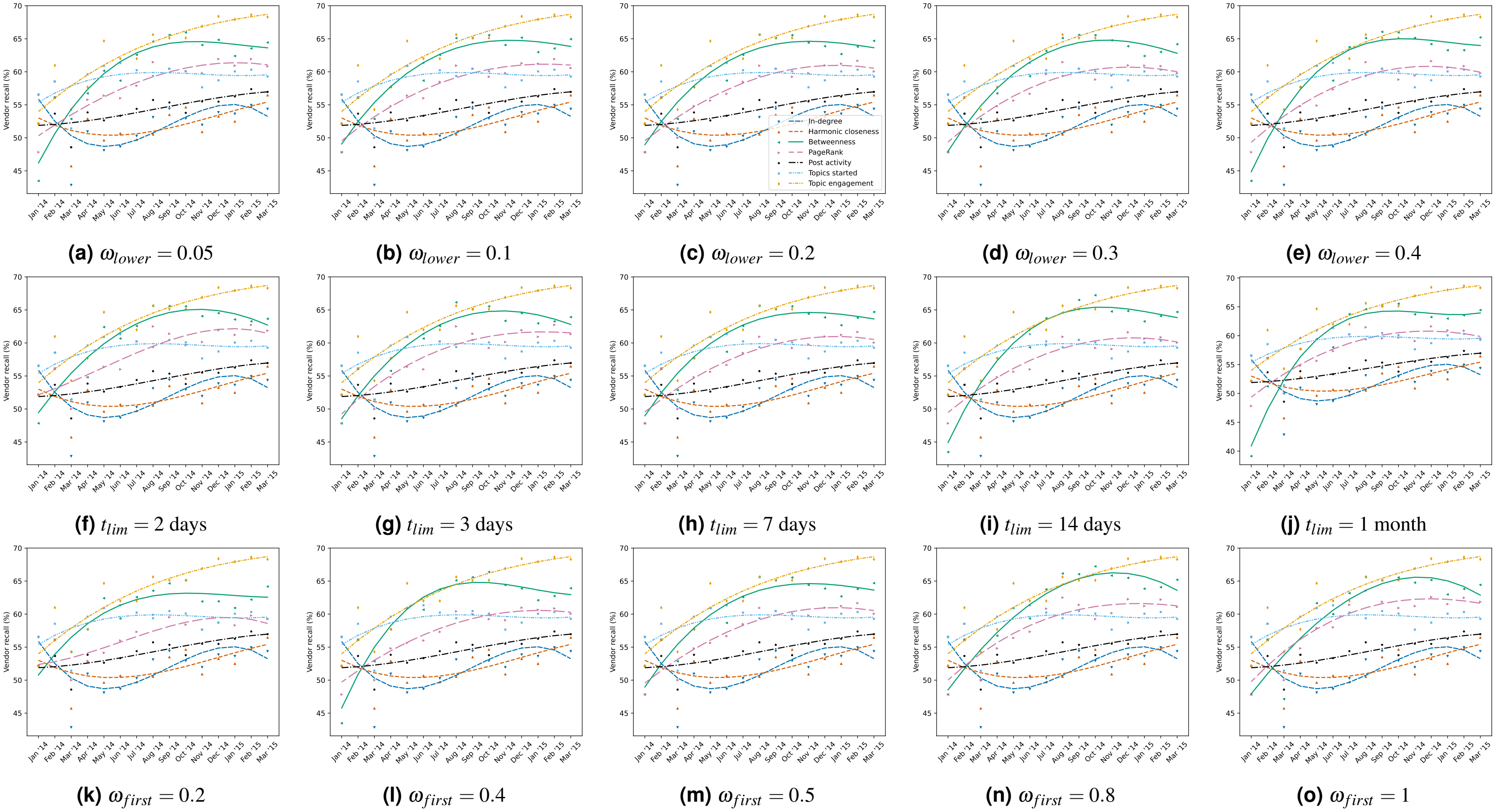

(a) $\omega_{lower} = 0.05$ **(b)** $\omega_{lower} = 0.1$ **(c)** $\omega_{lower} = 0.2$ **(d)** $\omega_{lower} = 0.3$ **(e)** $\omega_{lower} = 0.4$

(f) $t_{lim} = 2$ days **(g)** $t_{lim} = 3$ days **(h)** $t_{lim} = 7$ days **(i)** $t_{lim} = 14$ days **(j)** $t_{lim} = 1$ month

(k) $\omega_{first} = 0.2$ **(l)** $\omega_{first} = 0.4$ **(m)** $\omega_{first} = 0.5$ **(n)** $\omega_{first} = 0.8$ **(o)** $\omega_{first} = 1$

Figure S2. Results for varying edge weighting parameters. Monthly vendor recall of top vendor percentile (top 0-20% vendors in terms of sales) among the top 20% of all users based on the network measures and activity indicators for current success. Each plot displays monthly vendor recall for a different set of parameter values used for generating the network. Each row of plots varies a single parameter influencing edge weighting, with the remaining parameters at their default values. The center column always corresponds exactly with the default parameters.

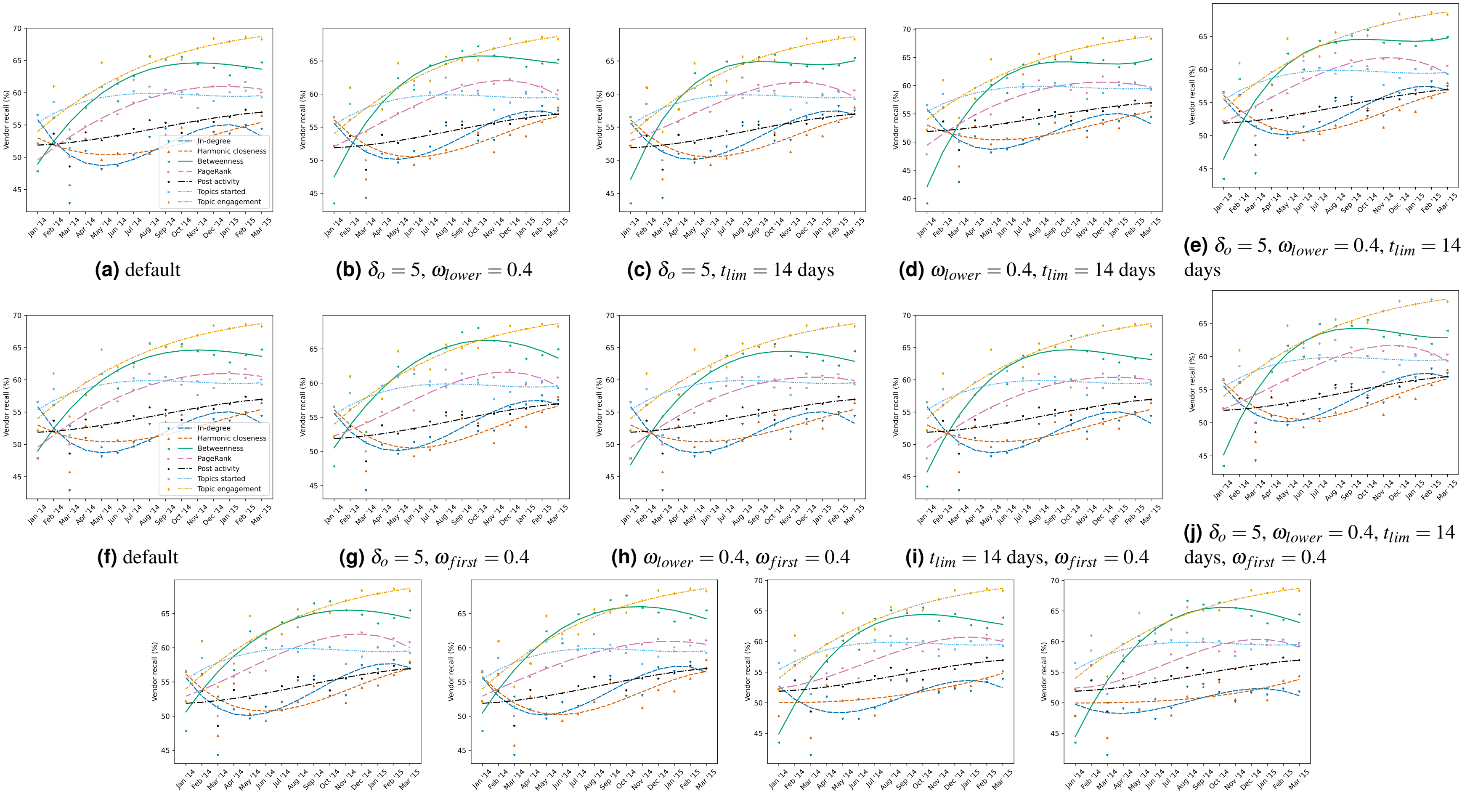

(a) default **(b)** $\delta_o = 5$, $\omega_{lower} = 0.4$ **(c)** $\delta_o = 5$, $t_{lim} = 14$ days **(d)** $\omega_{lower} = 0.4$, $t_{lim} = 14$ days **(e)** $\delta_o = 5$, $\omega_{lower} = 0.4$, $t_{lim} = 14$ days

(f) default **(g)** $\delta_o = 5$, $\omega_{first} = 0.4$ **(h)** $\omega_{lower} = 0.4$, $\omega_{first} = 0.4$ **(i)** $t_{lim} = 14$ days, $\omega_{first} = 0.4$ **(j)** $\delta_o = 5$, $\omega_{lower} = 0.4$, $t_{lim} = 14$ days, $\omega_{first} = 0.4$

(k) $\delta_o = 5$, $\delta_t = 14$ days **(l)** $\delta_o = 5$, $\delta_t = 3$ months **(m)** $\delta_o = 20$, $\delta_t = 14$ days **(n)** $\delta_o = 20$, $\delta_t = 3$ months

Figure S3. Results for varying multiple edge weighting parameters. Monthly vendor recall of top vendor percentile (top 0-20% vendors in terms of sales) among the top 20% of all users based on the network measures and activity indicators for current success. Each plot displays monthly vendor recall for a different set of parameter values used for generating the network. For the top two rows, each set of varied parameters consist of those that showed some improvement in Figures S1 and S2. The bottom row specifically compares different variations for δ_o and δ_t, to investigate the effects for different scopes of information loss.

S2 Post activity and Sales recall

In this section, we consider the *post activity recall* and *sales recall*, which measure respectively what percentage of post activity and sales of the entire top percentile the detected vendors are responsible for. We plot the monthly post activity recall in Figure S4 and current and future sales recall in Figure S5. These figures show the recall in terms of what percentage of post activity/sales the recalled vendors are responsible for.

From Figure S4 we see that the vast majority of post activity by the top vendor percentile is associated with vendors with high network centrality and activity indicators. As such, many of the non-recalled vendors are likely to be those with very few posts. Not unexpectedly, the post activity indicator often has the highest post activity recall, while the topics started indicator often captures the least post activity.

Figure S5 shows that for current success most of our observations for vendor recall hold up. Perhaps the most significant change is that the differences between PageRank and topics started and between betweenness centrality and topic engagement are more prominent. Similarly, for future success PageRank now outperforms the topics started baseline more consistently. For both current and future success, we observe that the sales recall is generally between 10-20% higher than the corresponding vendor recall. This indicates that the detected vendors are, on average, the more successful vendors among the top percentile.

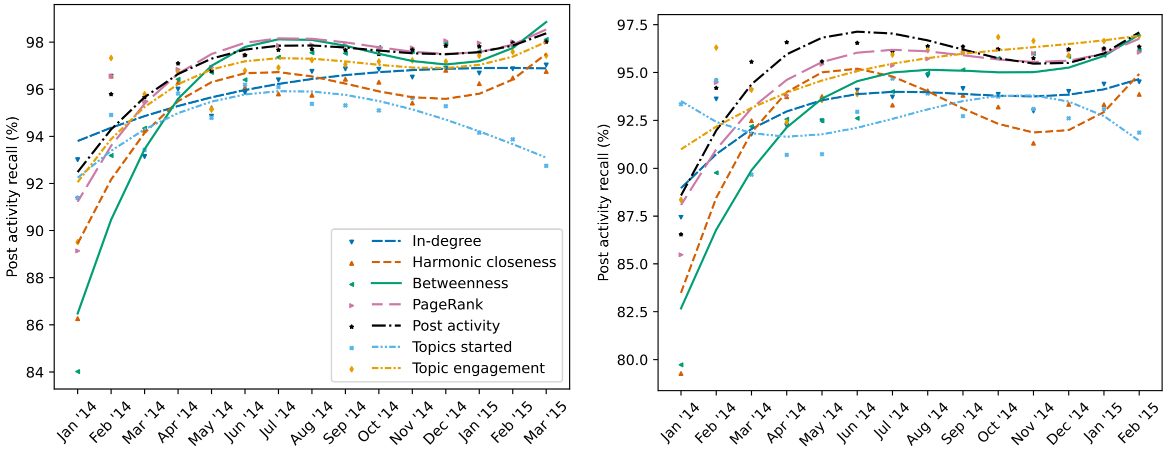

(a) Current success **(b)** Future success

Figure S4. Monthly post activity recall for both current (a) and future success (b). Higher post activity recall indicates that recalled vendors placed a relatively larger share of the top vendor percentile's total post activity.

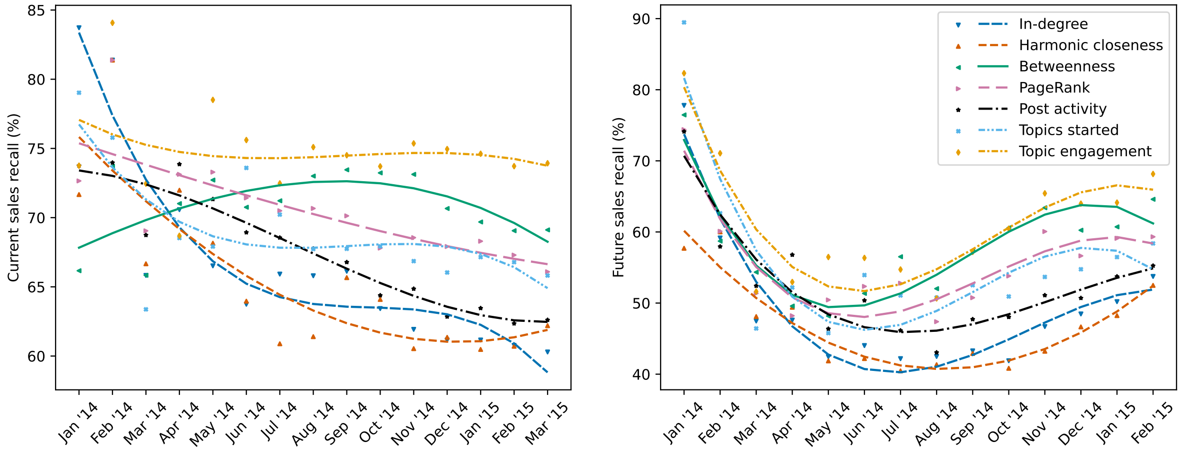

(a) Current success **(b)** Future success

Figure S5. Monthly sales recall for both current (a) and future success (b). Higher sales recall indicates a greater portion of the top vendor percentile's total sales was attributed to the recalled vendors.

S3 Overviews of sales and post activity of recalled and non-recalled users

Here, we explore the sales and post activity of recalled and non-recalled vendors for the various network centrality measures (see Figure S6) and months (see Figures S7, S8 and S9). We do so, to check if the conclusions drawn from the case study included in the main paper (Figure 3) are robust for the other months and if those conclusions also apply to the other centrality measures.

Figure S6 confirms that, for all centrality measures, there exists a threshold of activity (around 100 posts for September 2014) beyond which users are always included. Furthermore, we observe that in-degree, harmonic closeness centrality, and PageRank are far less inclined to include low activity vendors within the top 20% of their rankings. Although less obvious, for these centrality measures it does still appear to hold that greater success leads to greater odds of being included at lower activity. Whereas for betweenness centrality we found that the vendors uniquely found were relatively less active and more successful, we find that only the latter holds for the other measures. This lines up with our earlier finding from Table 1, that in-degree, harmonic closeness, and PageRank uniquely find very few vendors that are not likely to be found through their forum activity.

Figure S7 shows that, throughout all months, a threshold of activity exists beyond which users are always included. This threshold seems to slowly increase as the forum and the overall activity increases. However, after October 2014 this increase seems marginal at best. Furthermore, each month we observe the tendency for moderate activity vendors to be more likely to be included the more successful they are. We confirm this in Figure S8 by grouping vendors by their activity. Here, we have excluded the first few months which we in the main paper found to be a development period for the network, before achieving reliable vendor recall. We observe that indeed each month, though to a less significant extent for some, the average success of vendors uniquely found by betweenness exceeds that of those uniquely found by topic engagement. Furthermore, Figure S9 confirms our finding that this difference in average success is larger for future success. Thus, we can conclude that our findings in the main paper (with regards to Figure 3) are robust beyond only September 2014.

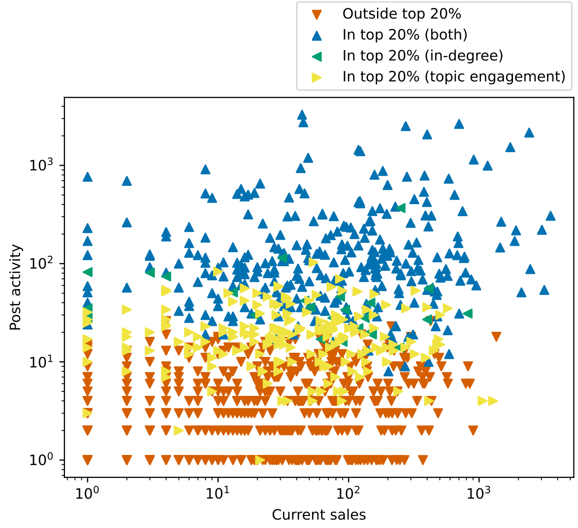

(a) In-degree

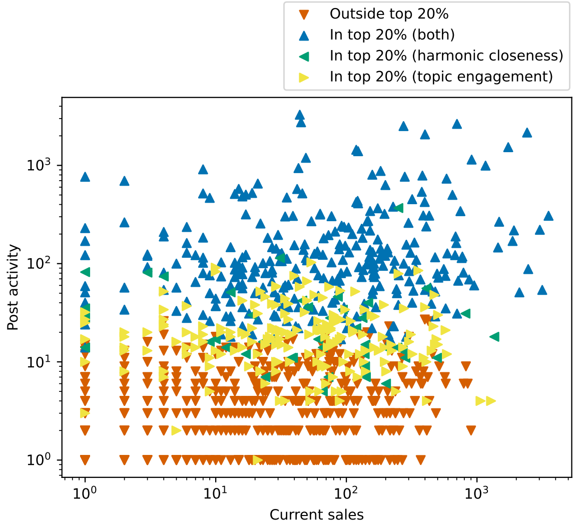

(b) Harmonic closeness centrality

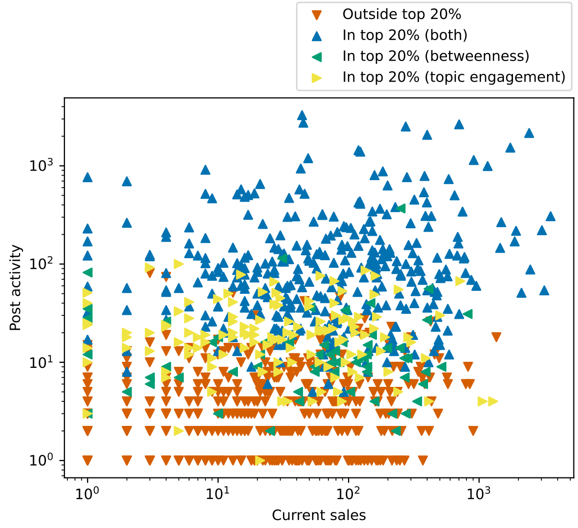

(c) Betweenness centrality

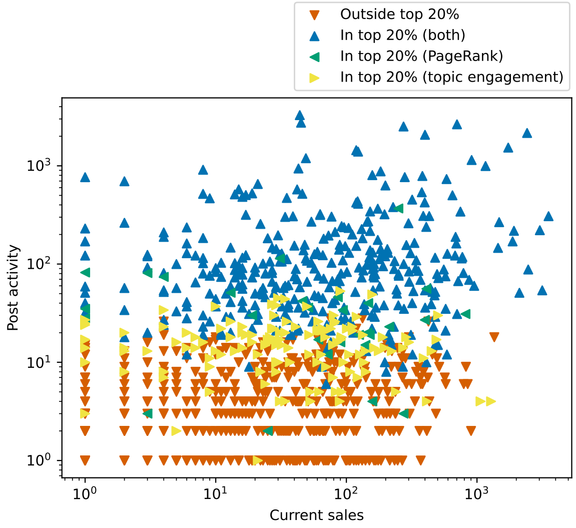

(d) PageRank

Figure S6. Sales and post activity of recalled (in top 20%) and non-recalled (outside top 20%) users for September 2014, for each of the network centrality measures compared with topic engagement and their intersection, respectively.

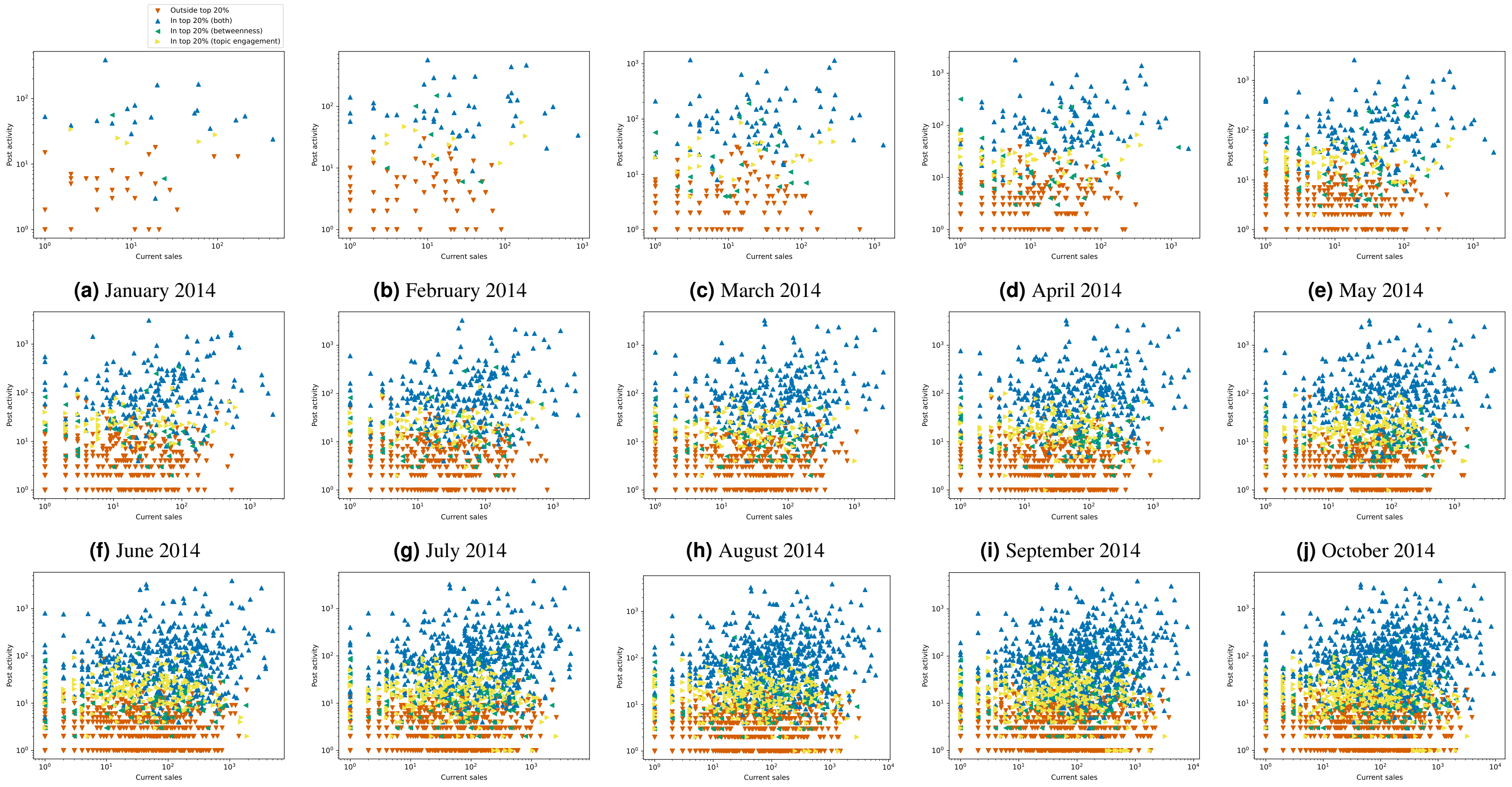

(a) January 2014 **(b)** February 2014 **(c)** March 2014 **(d)** April 2014 **(e)** May 2014

(f) June 2014 **(g)** July 2014 **(h)** August 2014 **(i)** September 2014 **(j)** October 2014

(k) November 2014 **(l)** December 2014 **(m)** January 2015 **(n)** February 2015 **(o)** March 2015

Figure S7. Current sales and post activity of recalled (in top 20%) and non-recalled (outside top 20%) users for topic engagement, betweenness centrality, and their intersection, for each month.

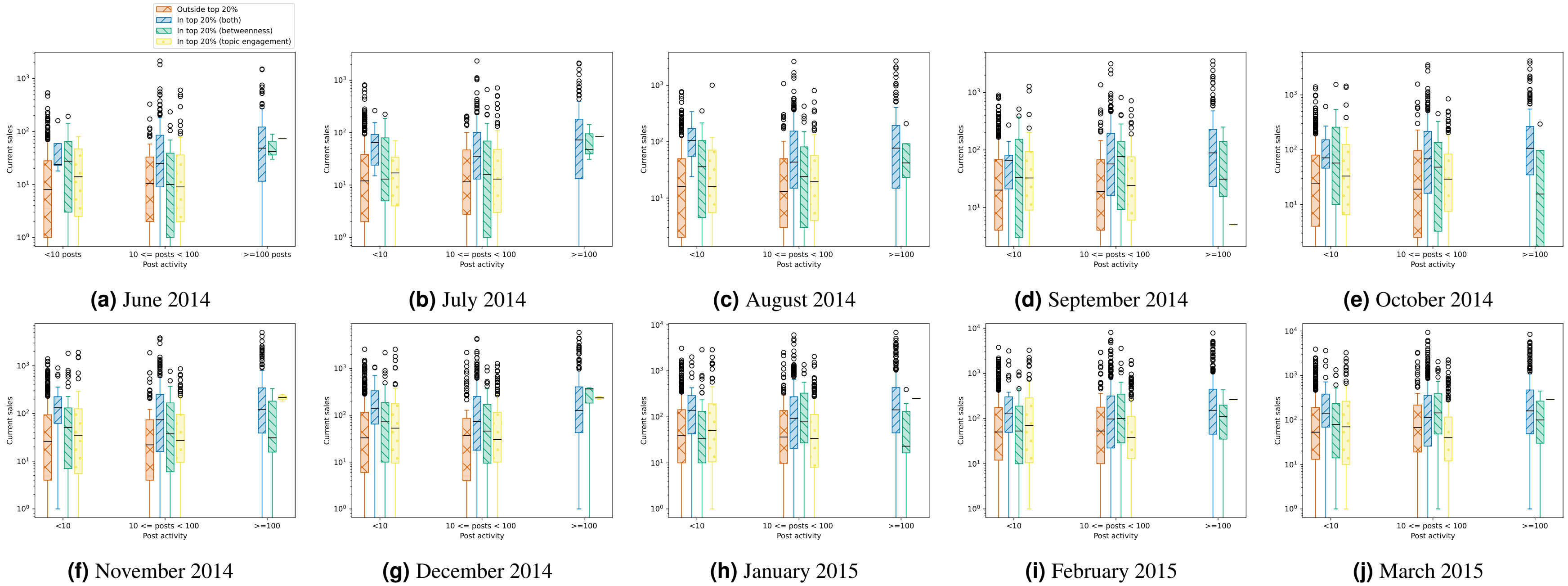

(a) June 2014 **(b)** July 2014 **(c)** August 2014 **(d)** September 2014 **(e)** October 2014

(f) November 2014 **(g)** December 2014 **(h)** January 2015 **(i)** February 2015 **(j)** March 2015

Figure S8. Current sales and post activity of recalled (in top 20%) and non-recalled (outside top 20%) users for topic engagement, betweenness centrality, and their intersection, for each month.

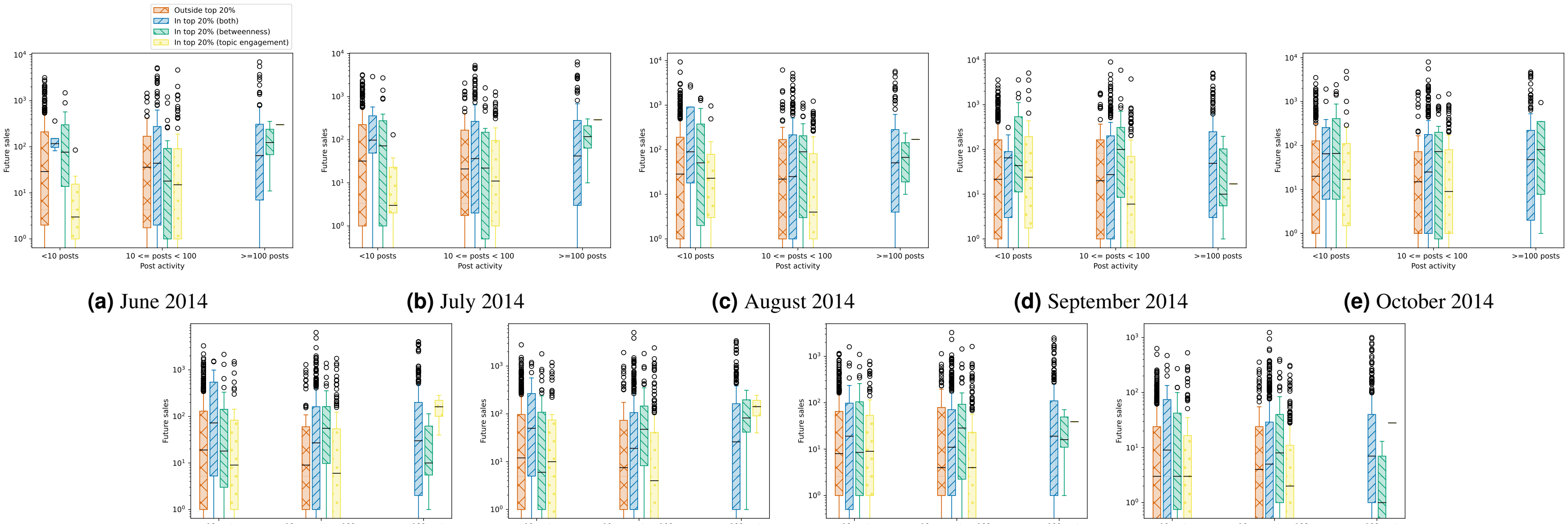

(a) June 2014 **(b)** July 2014 **(c)** August 2014 **(d)** September 2014 **(e)** October 2014

(f) November 2014 **(g)** December 2014 **(h)** January 2015 **(i)** February 2015

Figure S9. Current sales and post activity of recalled (in top 20%) and non-recalled (outside top 20%) users for topic engagement, betweenness centrality, and their intersection, for each month.

S4 Performance at different thresholds

The main paper presented evaluation metric results for a specific threshold for the measure rankings, namely 20% of all users. Here, we use ROC curves (receiver operating characteristic curve) to investigate the performance of the activity indicators and network measures at different thresholds. Specifically, we computed statistics at each interval of 5% in the range 0–100%, checking the performance for predicting groups of vendors for each of our measures. We consider the entire top vendor percentile, "less active" vendors among the top vendor percentile, all vendors, and all "less active" vendors. We set the activity threshold for "less active" at fewer than 100 posts, above which all vendors were shown to be found for all centrality measures. The resulting ROC curves, for September 2014, are shown in Figure S11.

For the top vendor percentile (Figures S11a,b), we see that topic engagement and betweenness centrality achieve a similar true positive rate up to a false positive rate of 20%. Afterwards, the topic engagement clearly outperforms all centrality measures. One factor contributing to the poor performance of betweenness centrality at false positive rates above 20%, is that many users get the same lowest betweenness value of zero. Since identically scoring users are essentially randomly ordered, their ordering does not add any predictive power. In Figure S10 we show that for each monthly snapshot around 70% of users end up with the same lowest value for betweenness centrality, while these percentages are far lower for the remaining measures. As such, betweenness is not suited for higher thresholds as they would include increasingly more users that are essentially randomly ordered. However in practice, given the limited resources of law enforcement, we are far more interested in the performance at low false positive rates. After all, the higher the false positive rate, the more resources would be wasted on non-vendors. As such, Figures S11a,b confirm that at least for any lower thresholds, our findings with regards to vendor recall hold up.

When we consider all vendors, not just the top vendor percentile, Figures S11c,d paint a different picture. Again, topic engagement provides the best overall performance, but now closely followed by the topics started indicator. On the contrary, though still outperforming the remaining measures at low thresholds, betweenness centrality has clearly worse performance. However, this is is in line with our conclusions, drawn from the results in the "Detecting vendors in the user base" section in the main paper, that topic engagement is the best overall predictor and that betweenness centrality performs particularly well for the more successful vendors. The performance of the topics started indicator for all vendors compared to for successful vendors, indicates that starting topics on the Evolution forum was quite indicative of being a vendor in general.

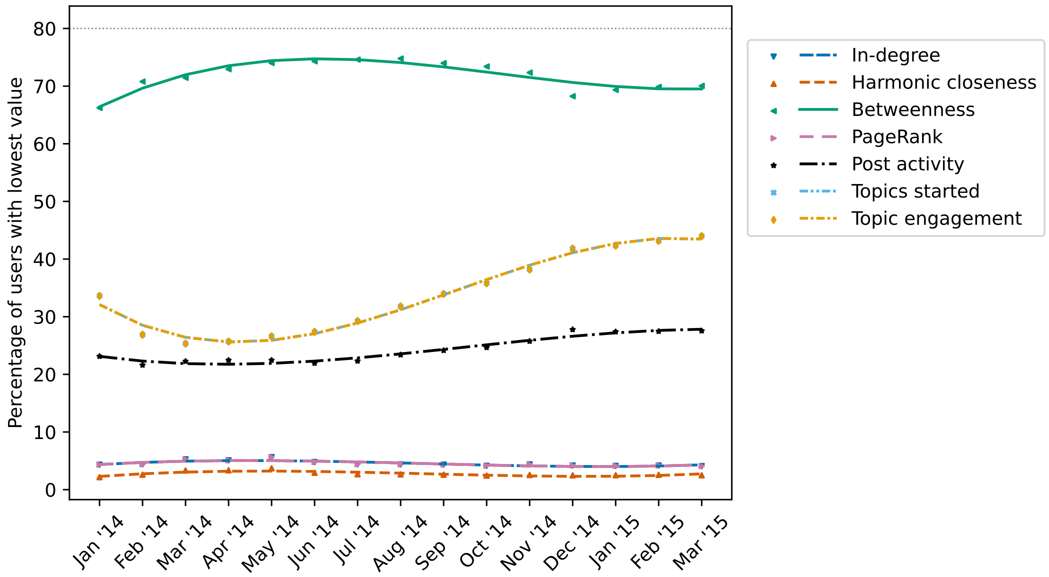

Figure S10. Percentage of users with the lowest value. Identical values indicate these users may have any random ordering, thus providing no additional predictive power. The dotted line at 80% indicates the threshold that would need to be exceeded for these random orderings to impact our standard threshold for the measure rankings of 20% of all users. None of the measures exceed this threshold for any of the snapshots.

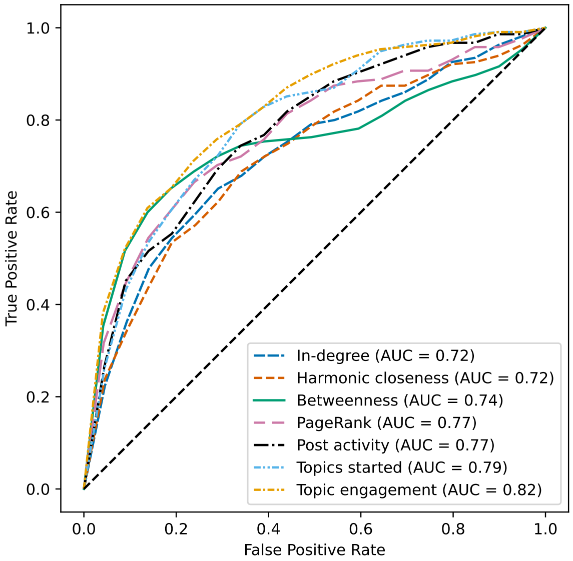

(a) Top percentile vendors regardless of activity.

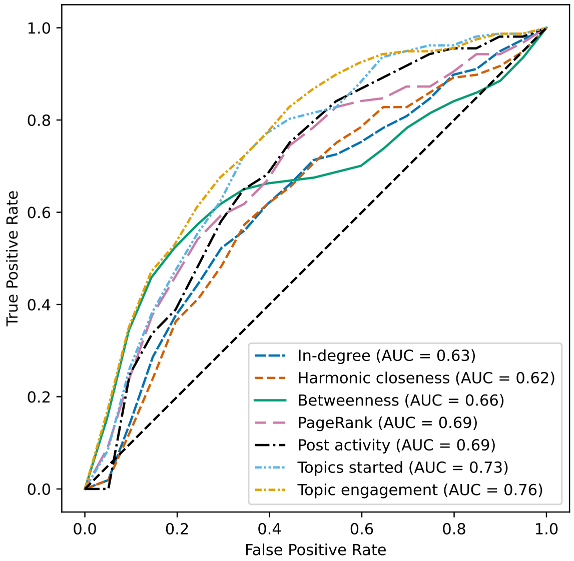

(b) Top percentile vendors with fewer than 100 posts

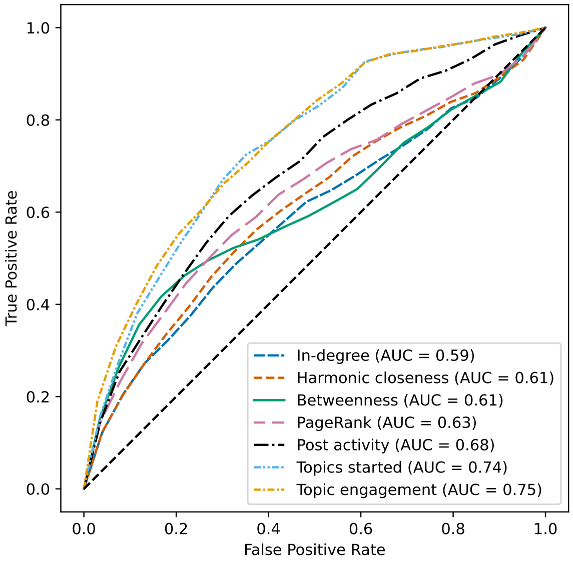

(c) All vendors regardless of activity.

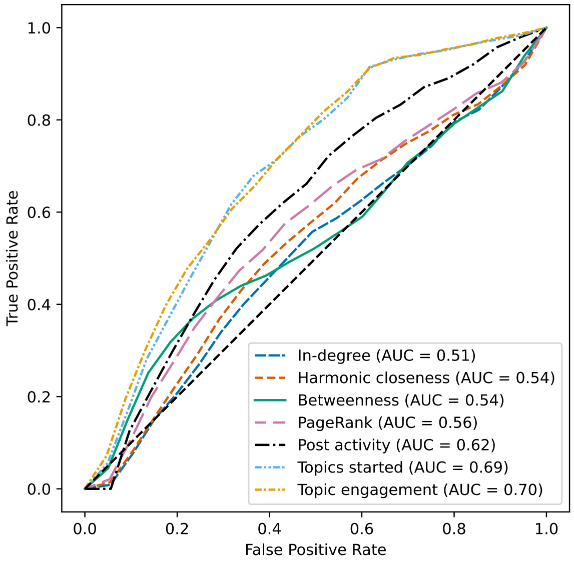

(d) All vendors with fewer than 100 posts

Figure S11. ROC curves for September 2014, predicting groups of vendors.
